# Methodological Considerations in Social Cost Studies of Addictive Substances: A Systematic Literature Review

**DOI:** 10.3389/fpubh.2016.00295

**Published:** 2017-01-18

**Authors:** Nick Verhaeghe, Delfine Lievens, Lieven Annemans, Freya Vander Laenen, Koen Putman

**Affiliations:** ^1^Department of Public Health, Interuniversity Centre for Health Economics Research (I-CHER), Vrije Universiteit Brussel, Brussels, Belgium; ^2^Department of Public Health, Interuniversity Centre for Health Economics Research (I-CHER), Ghent University, Ghent, Belgium; ^3^Institute for International Research on Criminal Policy (IRCP), Ghent University, Ghent, Belgium

**Keywords:** cost-of-illness, methodology, review, alcohol, tobacco, illicit drugs, psychoactive pharmaceuticals

## Abstract

**Background:**

Alcohol, tobacco, illicit drugs, and psychoactive pharmaceuticals’ use is associated with a higher likelihood of developing several diseases and injuries and, as a consequence, considerable health-care expenditures. There is yet a lack of consistent methodologies to estimate the economic impact of addictive substances to society. The aim was to assess the methodological approaches applied in social cost studies estimating the economic impact of alcohol, tobacco, illicit drugs, and psychoactive pharmaceuticals.

**Methods:**

A systematic literature review through the electronic databases, Medline (PubMed) and Web of Science, was performed. Studies in English published from 1997 examining the social costs of the addictive substances alcohol, tobacco, illicit drugs, and psychoactive pharmaceuticals were eligible for inclusion.

**Results:**

Twelve social cost studies met the inclusion criteria. In all studies, the direct and indirect costs were measured, but the intangible costs were seldom taken into account. A wide variety in cost items included across studies was observed. Sensitivity analyses to address the uncertainty around certain cost estimates were conducted in eight studies considered in the review.

**Conclusion:**

Differences in cost items included in cost-of-illness studies limit the comparison across studies. It is clear that it is difficult to deal with all consequences of substance use in cost-of-illness studies. Future social cost studies should be based on sound methodological principles in order to result in more reliable cost estimates of the economic burden of substance use.

## Introduction

The use and/or misuse of the addictive substances alcohol, tobacco, and illicit drugs are a worldwide problem contributing to the global burden of disease (1). Alcohol is responsible for 3.3 million deaths (5.9% of all deaths worldwide) each year and accounts for 5.1% of the global burden of disease (2). In 2015, tobacco smoking including second-hand smoking accounted for 7.2 million deaths (1), while for illicit drugs, this was 0.8% (3). In addition, the misuse of psychoactive pharmaceuticals such as antidepressants, sedatives, anxiolytics, and antipsychotics has also become a public health concern (4). The scale of the impact of the misuse of such pharmaceuticals worldwide remains, however, unknown due to a lack of epidemiological data. Nevertheless, a high prevalence of non-medical prescription drug use has been reported in countries such as the US, Canada, Australia, and some European countries (5, 6).

The use of alcohol, tobacco, illicit drugs, and psychoactive pharmaceuticals is associated with a considerable risk of developing a number of diseases and injuries (7, 8). So, it is clear that they affect the health and economic welfare of societies. The economic burden of these addictive substances can be estimated by cost-of-illness studies (9). In such studies, the social costs associated with a particular disease or condition are measured by estimating the direct, the indirect, and the intangible costs. The direct costs are those to deal with the disease, or condition, or its proximate effects (e.g., hospitalization and medication). The indirect costs are the costs related to lost human productivity (e.g., productivity losses due to morbidity or mortality). The intangible costs can be considered as non-financial welfare losses such as reduced health-related quality of life (9, 10).

In general, two approaches are used in cost-of-illness studies, namely, the prevalence-based and the incidence-based approaches. Prevalence-based studies estimate the costs associated with past and current consequences of the disease or condition in a given time period, typically a year. The incidence-based approach estimates the costs and consequences associated with new cases of the disease or condition in the current and future years (8). The indirect costs can be measured using the human capital method, the demographic method, and the friction cost method. The human capital method measures the current and future productivity losses occurring in the current year (10, 11). The demographic method measures the current costs from all current and past productivity losses by comparing the current population with a hypothetical population in which the disease or condition did not exist (10, 11). The friction cost method takes into account the productivity losses related to the time period until another employee takes over the work at the same capacity (12). The epidemiological concept of substance-attributable fractions (SAF) can be used to quantify the proportion of morbidity and mortality of diseases and conditions known to be causally related to substance use (13, 14).

Comparing the findings of social cost studies is difficult and must be cautiously interpreted due to differences in methodologies such as cost items included or calculation methods (15). Social cost studies are frequently characterized by some degree of uncertainty related to the availability and reliability of data sources (10). With the current review, it was the aim to evaluate the methodological approaches applied in social cost studies of the addictive substances alcohol, tobacco, illicit drugs, and psychoactive pharmaceuticals.

## Methods

**Figure 1:**
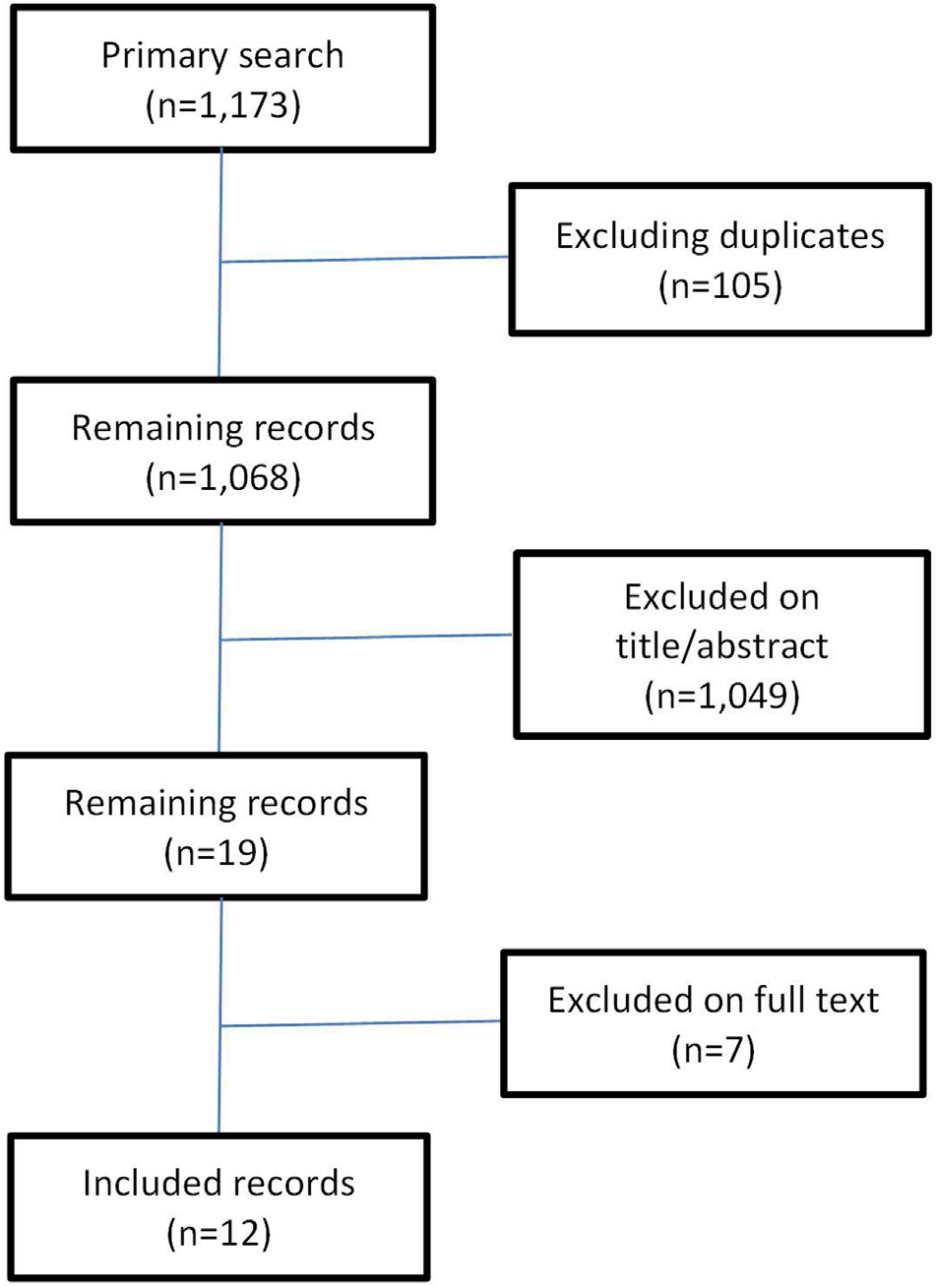
**Systematic literature review search process**.

A systematic literature search was conducted searching the electronic peer-reviewed databases, Medline (PubMed) and Web of Science. For each of the databases, a search algorithm was developed adapted to the specific requirements or features of the databases using the following entry terms: “cost-of-illness” (MeSH), “health-care costs” (MeSH), “cost,” “costs,” “social,” “societal,” “direct,” “indirect,” “intangible,” “alcohol,” “tobacco” (MeSH), “illicit,” “illegal,” and “psychotropic drugs” (MeSH). The initial search yielded 1,173 records. After excluding the duplicates (*n* = 15), 1,158 records remained for further evaluation (Figure 1). First, a selection on title and/or abstract was performed. Studies in English conducted in high-income Western countries estimating the social costs of the substances alcohol, tobacco, illicit drugs, and psychoactive pharmaceuticals were eligible for inclusion. In 1996, the first edition of the guidelines for estimating the costs of substance abuse was published (16). Therefore, for this review, studies published from January 1997 until December 2015 were considered. Studies were excluded if they consisted of health economic evaluations of substance abuse treatment or prevention programs, or if the geography, language, and time period criteria were different from those described under the inclusion criteria. The selection on title and/or abstract resulted in 19 records of which the full text was evaluated in detail on the inclusion and exclusion criteria. Seven records were excluded resulting in 12 social cost studies included in the review. The outcomes of interest included the substance under study, the cost measurement approach (incidence-based or prevalence-based), the major cost categories considered (direct, indirect, and intangible costs), productivity losses measurement (human capital, demographic, or friction cost measurement), cost items considered, and a number of reporting issues. Study quality was assessed using a checklist for social cost studies (Table A1 in Appendix) (17).

## Results

The literature review encompassed 12 studies. Five of them were conducted in Germany (18–22), while the remaining were from Canada (23), Denmark (24), France (25), Scotland (26), Spain (27), Sweden (28), and the US (29). In two studies (23, 25), the social costs of alcohol, tobacco, and illicit drugs were estimated, while in the other studies, the economic impact of one particular substance was considered (Table 1). A prevalence-based approach was used in 11 of 12 studies (Table 1). In all studies, both the indirect and the direct cost categories were accounted for, while in only two studies (19, 28), also welfare losses were considered. In Jarl et al. (28), quality of life of alcohol consumers, their family, and friends was taken into account by calculating the number of quality-adjusted life years (QALYs) for the consumers and a weighted quality of life estimate for the relatives. In Konnopka and König (19), also QALYs were used to express the impact of alcohol on quality of life of individuals consuming moderate alcohol levels. In both studies in the base case analysis, no monetary valuation of the alcohol-attributable welfare losses was included. The human capital approach was used in all studies to estimate the indirect costs associated with substance use (Table 1).

**Table 1:** **Overview of social cost studies included in the literature review**.

Reference	Country	Substance	Cost categories	Cost measurement	Productivity losses measurement
Ruff et al. (21)	Germany	Tobacco	Direct costs/indirect costs	Prevalence-based	Human capital method
Garcia-Altes et al. (27)	Spain	Illicit drugs	Direct costs/indirect costs	Prevalence-based	Human capital method
Varney and Guest (26)	Scotland	Alcohol	Direct costs/indirect costs	Prevalence-based	Human capital method
Fenoglio et al. (25)	France	Alcohol, tobacco, and illicit drugs	Direct costs/indirect costs	Prevalence-based	Human capital method
Rasmussen et al. (24)	Denmark	Tobacco	Direct costs/indirect costs	Incidence-based	Human capital method
Neubauer et al. (20)	Germany	Tobacco	Direct costs/indirect costs	Prevalence-based	Human capital method
Konnopka and König (18)	Germany	Alcohol	Direct costs/indirect costs	Prevalence-based	Human capital method
Rehm et al. (23)	Canada	Alcohol, tobacco, and illicit drugs	Direct costs/indirect costs	Prevalence-based	Human capital method
Jarl et al. (28)	Sweden	Alcohol	Direct costs/indirect costs/intangible costs	Prevalence-based	Human capital method
Konnopka et al. (33)	Germany	Alcohol	Direct costs/indirect costs/intangible costs	Prevalence-based	Human capital method
Hansen et al. (29)	US	Psychoactive pharmaceuticals	Direct costs/indirect costs	Prevalence-based	Human capital method
Wacker et al. (22)	Germany	Tobacco	Direct costs/indirect costs	Prevalence-based	Human capital method

Information on the total costs as a proportion of the gross domestic product (GDP) was provided in four studies (Table 2). The quantification of the amount of morbidity and mortality that could be attributed to legal or illegal drugs occurred in seven studies by applying the concept of SAFs (Table 2). In all seven studies, information on the input parameters to calculate the SAFs was provided. In the study by Ruff et al. (21), attributable risks for tobacco-associated diseases were applied, but no information on the data source was provided. In Garcia-Altes et al. (27), the estimation of the attributable risks was based on the findings from previous studies and on Spanish statistical data. Sensitivity analyses were conducted in eight studies (Table 2). In general, three categories of sensitivity analyses could be distinguished (Table 3). A first category was related to the use of alternative methodological approaches. In five studies (18, 20, 22, 23, 28), this consisted of applying the friction cost method as an alternative method to estimate the substance-attributable indirect costs. In one study (28), as a sensitivity analysis, the welfare losses—expressed as QALYs—were valued. A second category included sensitivity analyses related to the inclusion or exclusion of certain cost items (Table 3). In a third category of sensitivity analyses, input parameters such as the relative risks of the substance-attributable diseases or resource use were varied (Table 3).

**Table 2:** **Reporting issues of the studies included in the review**.

Reference	Substance	Details on cost items	Sensitivity analyses	% of GDP	SAF
Ruff et al. (21)	Tobacco	Disaggregated	No	No	Not clear
Garcia-Altes et al. (27)	Illicit drugs	Disaggregated	No	Yes	No
Varney and Guest (26)	Alcohol	Disaggregated	Yes	No	No
Fenoglio et al. (25)	Alcohol/tobacco/illicit drugs	Disaggregated	No	Yes	Yes
Rasmussen et al. (24)	Tobacco	Aggregated	Yes	No	Yes
Neubauer et al. (20)	Tobacco	Disaggregated	Yes	No	Yes
Konnopka and König (18)	Alcohol	Disaggregated	Yes	Yes	Yes
Rehm et al. (23)	Alcohol/tobacco/illicit drugs	Disaggregated	Yes	No	Yes
Jarl et al. (28)	Alcohol	Disaggregated	Yes	Yes	Yes
Konnopka et al. (33)	Alcohol	Disaggregated	Yes	No	Yes
Hansen et al. (29)	Psychoactive pharmaceuticals	Disaggregated	No	No	No
Wacker et al. (22)	Tobacco	Disaggregated	Yes	No	No

*SAF, substance-attributable fraction; GDP, gross domestic product*.

**Table 3:** **Applied sensitivity analyses in the studies included in the review**.

Category	Type	Reference
Methodological approaches	Friction cost method	Wacker et al. (22), Jarl et al. (28), Konnopka and König (18), Rehm et al. (23), and Neubauer et al. (20)
	Valuation of QALYs	Jarl et al. (28)
Cost items	Exclusion of unpaid work	Konnopka et al. (33), Konnopka and König (18)
	Inclusion of unpaid work	Neubauer et al. (20)
Input parameters	Resource use	Wacker et al. (22), Jarl et al. (28), and Varney and Guest (26)
	Relative risks	Konnopka et al. (33) and Rasmussen et al. (24)
	Substance consumption rates	Konnopka et al. (33)
	Discount rates	Konnopka et al. (33), Konnopka and König (18), and Rasmussen et al. (24)

*QALYs, quality-adjusted life years*.

In 11 of 12 studies, the major cost categories “direct costs” and “indirect costs” were reported in a disaggregated form (Table 2). This means that, for each cost item, detailed information on the costs was provided. Contrary, in the study by Rasmussen et al. (24), only the total direct and indirect tobacco-attributable costs were reported. For the direct costs, substance-attributable hospitalization costs were included in all studies, followed by pharmaceuticals (*n* = 11), and primary care costs (*n* = 9). Contrary, a number of cost items such as accident and emergency care, laboratory tests, home-based nursing care, and household care were accounted for in only a limited number of studies (Table 4). An important reason for not considering certain cost items was the absence of accurate and reliable data. For the indirect costs, disability/absenteeism and premature mortality were accounted for in 11 studies each, while substance-attributable costs related to unemployment were included in only two studies (Table 4).

**Table 4:** **Cost items pertaining to the major cost categories included in the social cost studies**.

Cost items	Ruff et al. (21)	Garcia-Altes et al. (27)	Varney and Guest (26)	Fenoglio et al. (25)	Rasmussen et al. (24)	Neubauer et al. (20)	Konnopka and König (18)	Rehm et al. (23)	Jarl et al. (28)	Konnopka et al. (33)	Hansen et al. (29)	Wacker et al. (22)
**Direct costs**												
Hospitalization	x	x	x	x	x	x	x	x	x	x	x	x
A&E		x	x									
Ambulatory care	x		x		x	x	x	x			x	x
Ambulance		x	x	x								
Residential care				x				x		x		
Rehabilitation	x					x	x			x		x
Pharmaceuticals	x	x	x	x	x	x	x	x	x	x		x
Primary care		x	x	x	x	x			x	x	x	x
Laboratory tests			x									
Home-based nursing care	x	x										
Social services			x						x			
Household care										x		
Non-medical costs							x					
Education		x								x		
Prevention		x	x	x				x	x	x		
Research		x		x				x	x	x		
**Indirect costs**												
Disability/absenteeism	x	x	x	x	x	x	x	x	x	x		x
Unemployment			x								x	
Premature mortality	x	x	x	x	x	x	x	x	x	x	x	
Early retirement	x				x	x	x		x	x		
**Intangible costs**												
QALY									x	x		

*A&E, Accident and Emergency Department; QALY, quality-adjusted life year*.

As an example to illustrate how differences in cost items included may have affected the cost outcomes, we focus on the five studies conducted in Germany (18–22). For these studies, a more in-depth analysis of differences in cost items considered was performed. Certain cost items such as hospitalization, rehabilitation, and medication use were included in all five studies. Others were only considered in one particular study such as substance-attributable costs for home-based nursing care (21), household care (19), or non-medical costs (18) (Table 4). For the indirect costs, expenditures associated with disability, premature mortality, and early retirement were accounted for in four of five studies (18–21). In the study by Wacker et al. (22), only indirect costs due to disability were included in the analysis.

## Discussion

The aim of the current literature review was to evaluate the methodological approaches and considerations in studies examining the economic impact of alcohol, tobacco, illicit drugs, and psychoactive pharmaceuticals. In 11 of 12 studies included in the review, the prevalence-based approach was used. The choice for a prevalence-based or an incidence-based method is depending on the aim of the study. A prevalence-based approach is more appropriate for estimating the economic burden of a substance in a specified time period. The incidence-based approach portrays the magnitude of the economic impact during an individuals’ life course, thus providing insights into the value of preventing a case of substance use (8). In all studies, the major cost categories such as direct costs and indirect costs were included, while the intangible costs were accounted for in only two studies. The latter costs are often ignored in social cost studies of substance misuse since it is difficult to place a monetary value upon welfare losses (30). Considerable differences were found related to the cost items included or excluded across the studies in the review. Our findings are similar with those of a previous literature review, however, limited to social cost studies examining the economic burden of alcohol. Differences in methodologies related to the availability and accuracy of data were found to be important reasons explaining the differences in cost estimates (31). Our review was extended to all addictive substances. We found not only differences in method but also differences in the drug under study, with only limited studies examining more than one substance. It is clear that methodological inconsistencies have important effects putting the reliability of cost-of-illness findings into question. This may result in an underestimation or overestimation of the real economic burden of substance misuse to society (32). This is a critical element since the findings of such studies may serve as the basis for comparative health economic evaluations or for policy decisions (8). Methodological considerations are not limited to cost-of-illness studies of substance use, but they were also found in several literature reviews of the economic impact of mental disorders (33–35). For example, in their review, Luppa et al. (34) found that costs of morbidity and mortality were included in only half and in one-third of studies examining the social costs of depression.

It is thus clear that comparing the findings of social cost studies is difficult. A possible basis for comparing the findings across studies is presenting them relative to a country’s GDP. This occurred in four studies included in the current review. Nevertheless, even if a uniform methodology was to be developed and used, cross-country comparisons would necessitate sufficient contextualization since countries differ in terms of social security systems, institutional structures, and cultural traditions (36). In 11 of 12 studies included in the review, information on the relative proportion of the different cost items to the total costs was provided. This is important because different stakeholders may be interested in different outcomes. For governments, the findings can assist them in their decisions related to the funding of interventions designed to reduce the burden of substance misuse. Information regarding the impact of substance misuse on productivity can be useful for employers, while for households, the impact on medical or other expenses can be relevant (15, 16). Sensitivity analyses were applied in eight studies included in the review. Uncertainty around certain cost estimates in cost-of-illness studies is almost self-evident. It is, however, necessary to address this and inform the reader about the amount of uncertainty associated with the cost estimate outcomes (10).

Some limitations of our review need to be addressed. First, no robust analysis of the magnitude of the economic impact in monetary terms was performed, since we focused on methodological issues of social cost studies of substance use. Nevertheless, as an example, the influence of methodological choices on the cost outcomes for the German studies was examined. Second, in a number of studies (25–29), also drug-attributable crime and law enforcement costs were estimated. For the current review, methodological considerations related to the estimation of these costs were not considered. It is yet important to not omit these costs in studies examining the economic impact of substance use, since they may account for a considerable part of the total costs (23, 37, 38). For example, in the study by Rehm et al. (23) examining the social costs of alcohol, tobacco, and illicit drugs, the costs for law enforcement constituted more than one-third of the direct costs. Third, only studies published after the introduction of the guidelines for estimating the costs of substance abuse in 1996 were eligible for inclusion in the review. It is thus possible that we missed some relevant studies prior to 1997. Fourth, we did not search the gray literature. It is thus possible that we have missed social cost studies of addictive substances not published in peer-reviewed journals. Fifth, the current review was limited to social cost studies conducted in high-income countries. However, the economic burden attributable to the addictive substances alcohol, tobacco, and illicit drugs is also considerable in low- and middle-income countries (39). So, it is clear that addictive substances pose a considerable economic impact to societies worldwide. In conclusion, the current review has illustrated the complexity of dealing with all consequences of substance use in cost-of-illness studies. Future social cost studies should be based on sound methodological principles in order to result in more reliable cost estimates of the economic burden of substance use. This is important since the findings of such studies may be used as the basis for comparative health economic evaluations and for substance use policies.

## Author Contributions

NV contributed to the development of the study, conducted the literature search, and drafted the manuscript. KP participated in the development of the methods and reviewed the manuscript. LA, DL, and FL revised the manuscript. All the authors approved the final version of the manuscript.

## Conflict of Interest Statement

The authors declare that the research was conducted in the absence of any commercial or financial relationships that could be construed as a potential conflict of interest.

## Appendix

**Table A1:** **Quality appraisal of studies included in the literature review**.

Quality check item	Ruff (21)	Garcia-Altes et al. (27)	Varney and Guest (26)	Fenoglio et al. (25)	Rasmussen et al. (24)	Neubauer et al. (20)	Konnopk and König (18)	Rehm et al. (23)	Jarl et al. (28)	Konnopka et al. (33)	Hansen et al. (29)	Wacker et al. (22)
Clear definition of the illness?	x	x	x	x	x	x	x	x	x	x	x	x
Epidemiological sources carefully described?	x	x	x	x	x	x	x	x	x	x	x	x
Costs sufficiently disaggregated?	x	x	x	x		x	x	x	x	x	x	x
Activity data sources carefully described?	x	x	x	x	x	x	x	x	x	x	x	x
Activity data appropriately assessed?			x				x			x		x
Sources of all cost values analytically described?		x	x	x	x	x	x	x	x	x	x	x
Methods carefully explained?		x	x	x		x	x	x	x	x	x	
Costs discounted?	x					x	x	x	x	x		x
Major assumptions tested in a sensitivity analysis?			x		x	x	x	x	x	x		x
Presentation of results consistent with methodology?	x	x	x	x	x	x	x	x	x	x	x	x
